# Frontal Anatomical Correlates of Cognitive and Speech Motor Deficits in Amyotrophic Lateral Sclerosis

**DOI:** 10.1155/2019/9518309

**Published:** 2019-03-13

**Authors:** Yana Yunusova, Jamal Ansari, Joel Ramirez, Sanjana Shellikeri, Greg J. Stanisz, Sandra E. Black, Susan M. Gillingham, Alex Kiss, Donald T. Stuss, Lorne Zinman

**Affiliations:** ^1^University of Toronto, Department of Speech Language Pathology, Rehabilitation Sciences Institute, Toronto, Canada; ^2^Sunnybrook Research Institute, Hurvitz Brain Sciences Program, Toronto, Canada; ^3^University Health Network-Toronto Rehabilitation Institute, Toronto, Canada; ^4^L.C. Campbell Cognitive Neurology Research Unit, Sunnybrook Research Institute, University of Toronto, Toronto, Canada; ^5^Sunnybrook Health Sciences Centre and University of Toronto, Department of Medicine, Neurology, Toronto, Canada; ^6^Rotman Research Institute, Baycrest, Toronto, Canada; ^7^University of Toronto, Department of Psychology, Toronto, Canada

## Abstract

The goal of this study was to identify neurostructural frontal lobe correlates of cognitive and speaking rate changes in amyotrophic lateral sclerosis (ALS). 17 patients diagnosed with ALS and 12 matched controls underwent clinical, bulbar, and neuropsychological assessment and structural neuroimaging. Neuropsychological testing was performed via a novel computerized frontal battery (ALS-CFB), based on a validated theoretical model of frontal lobe functions, and focused on testing energization, executive function, emotion processing, theory of mind, and behavioral inhibition via antisaccades. The measure of speaking rate represented bulbar motor changes. Neuroanatomical assessment was performed using volumetric analyses focused on frontal lobe regions, postcentral gyrus, and occipital lobes as controls. Partial least square regressions (PLS) were used to predict behavioral (cognitive and speech rate) outcomes using volumetric measures. The data supported the overall hypothesis that distinct behavioral changes in cognition and speaking rate in ALS were related to specific regional neurostructural brain changes. These changes did not support a notion of a general dysexecutive syndrome in ALS. The observed specificity of behavior-brain changes can begin to provide a framework for subtyping of ALS. The data also support a more integrative framework for clinical assessment of frontal lobe functioning in ALS, which requires both behavioral testing and neuroimaging.

## 1. Introduction

Amyotrophic lateral sclerosis (ALS) is a multisystem disorder characterized by neurodegeneration of upper and lower motor neurons as well as extramotor pathways [1]. ALS is a rapidly progressing and highly debilitating condition, with motor neurodegeneration impacting both spinal (i.e., arm, trunk, and leg) and bulbar (i.e., speech and swallowing) musculatures. Bulbar motor signs and symptoms are associated with a disease phenotype with shorter survival and an overall more debilitating course, including higher association with upper motor neuron dysfunction and extramotor deficits [2–4]. Extramotor impairments in ALS impact cognitive functions across multiple domains including executive functions, social cognition, language, and memory. Most commonly, the profile of behavioral changes in ALS has been labelled as the “frontal dysexecutive syndrome” [5–8]. Yet, limited knowledge exists systematically linking clinical symptomology to the underlying neurostructural changes.

Among the existing frontal lobe models [9, 10], the model by Stuss and colleagues is characterized by high neuroanatomic specificity [11–15]. Burgess and Stuss argue that “what has been historically considered the executive system consists of multiple subsystems, with anatomical and behavioral separation” ([11], p. 763). In their model, all cognitive processes are driven by energization defined as the function of initiating and sustaining a response and represented uniquely in the superior medial brain regions (Brodmann areas 24, 9, and 6), bilaterally. The executive function encompasses basic attentional control processes of task setting (i.e., setting a stimulus-response relationship, planning) controlled by left lateral frontal regions and monitoring (i.e., maintaining quality of response over time, checking) controlled by the right lateral frontal regions. Emotional regulation is localized to the orbitomedial prefrontal cortex, and theory of mind and metacognition are linked to the more rostral prefrontal region (area 10). Inhibition is viewed by Stuss and colleagues [14, 15] as a descriptive psychological construct rather than an independent process; i.e., they have demonstrated on “inhibitory” tasks such as various versions of the Stroop that performance on these tasks can be deconstructed into different processes with different anatomical localizations within the frontal lobes [16–20]. When “inhibitory” tasks such as antisaccades are used and deconstruction has not been attempted or achieved, the relationship to the frontal lobes is described as a frontostriatal network, involving several frontal regions including the inferior frontal gyrus on the right, dorsolateral prefrontal cortex (DLPFC), frontal eye fields, and presupplementary motor areas [21, 22]. Identification of the neuroanatomic correlates of the specific frontal lobe processes has been elucidated through extensive neuropsychological testing of patients with well-defined focal lesions validated through structural neuroimaging [12, 19, 23–25] and verified in healthy controls using functional MRI and event-related potentials [26, 27].

The model by Stuss and colleagues and their testing and data interpretation procedures are highly applicable to the study of ALS since similar behavioral and neurostructural changes have been described in this disease. Behaviorally, the following impairments have been described in ALS patients: changes in attentional processes (including selective attention and attentional focus) [28, 29], energization deficit [30], facial recognition of emotions, memory for emotional words, and judgment of emotional states of others [31–35], as well as both cognitive and affective theory of mind (ToM) [32, 36, 37]. Antisaccades also showed high sensitivity to cognitive impairment in ALS [38–40]. Only a limited number of studies on social cognition, executive function, and antisaccades linked the results of neuropsychological testing to changes in brain structure and function [41–45].

In addition to the cognitive processes, speech production relies heavily on the integrity of anatomic-motor and extramotor regions within the frontal lobes [46, 47]. In a recent study that linked speech measures to metrics of brain structural integrity in a subtype of frontotemporal dementia (FTD) known as primary progressive aphasia (PPA), reduction in speaking rate was associated with atrophy in the inferior frontal region, supplementary motor area, and ventral precentral gyrus on the left [48]. Although linked to the cognitive-linguistic deficits in PPA and other types of aphasia [49], speaking rate is highly sensitive to bulbar motor changes in ALS [50]; however, its neuroanatomic correlates have not been identified.

The primary goal of this study was to identify the neuroanatomical correlates of cognitive and speaking rate abnormalities in ALS in a context of a prominent frontal lobe model [14]. Cognitive deficits were assessed using a novel ALS computerized frontal battery (ALS-CFB), developed by our group. ALS-CFB was designed to focus on the core of four frontal cognitive processes (e.g., energization, attention, emotion regulation, and metacognition); a computerized antisaccade task was also included [51]. Gillingham et al. reported selective deficits in energization, attention, and antisaccades in our ALS cohort. Here, we expanded our initial study to examine neurostructural underpinnings of the selected cognitive processes and speaking rate. We predicted that changes in the cognitive and speech performance will be linked to volumetric structural changes in the specific regions of the frontal lobes: (1) energization: superior medial regions bilaterally; (2) attentional: right and left lateral frontal regions [12, 15, 24]; (3) emotion regulation: to the orbitofrontal/ventral medial prefrontal cortex; and (4) metacognition: to the rostral prefrontal region, both bilaterally [14]. Further, (5) the performance on the antisaccades will be associated with the inferior frontal gyrus on the right and bilateral dorsolateral prefrontal cortex (DLPFC), frontal eye fields, and presupplementary motor areas [21, 22] and (6) speaking rate: mainly to the primary and secondary/associated motor areas including primary motor and somatosensory cortex and supplementary and presupplementary motor areas bilaterally as well as the left inferior frontal region, involved in the higher-order processes of facilitation and integration [52].

## 2. Methods

### 2.1. Participants

The study was approved by the Research Ethics Boards of Sunnybrook Health Science Centre. Participants provided informed consent according to the Declaration of Helsinki. Data from seventeen patients (*M* = 8, *F* = 9) diagnosed with ALS based on the revised El Escorial criteria [53] and twelve neurologically and cognitively intact controls (*M* = 6, *F* = 6) were used in the study. All participants passed the Montreal Cognitive Assessment (MoCA) [54]. They were all right-handed and able to complete both imaging and clinical portions of the testing. Patients were excluded at recruitment if they showed forced vital capacity below 80% and/or reported depression or prescription of antidepressant medications. The healthy controls were age- and education-matched to the patient group (see Table 1).

The demographic and clinical characteristics of participants are shown in Table 1. Thirteen patients presented with spinal onset of ALS; four patients reported disease onset in the bulbar musculature. Disease duration was calculated from the time of symptom onset.

### 2.2. Procedures

#### 2.2.1. Clinical and Bulbar Motor Testing

The impact of ALS on daily functions was documented using the ALS Functional Rating Scale-Revised questionnaire (ALSFRS-R) [55]. ALSFRS-R bulbar subscore was calculated based on 3 questions regarding speech, swallowing, and salivation functions. The upper motor neuron (UMN) involvement score, reflective of cortical and subcortical motor pathway abnormalities, was calculated based on the assessment of increased tone (0 = normal, 1 = increased), exaggerated reflexes (0 = absent, 2 = normal, 3 = brisk, and 4 = very brisk), pseudobulbar affect (0 = normal, 1 = present), and spastic dysarthria (0 = normal, 4 = severe).

Bulbar motor assessment was performed using the Sentence Intelligibility Test (SIT; [56]), which provided a measure of speaking rate (words per minute (WPM)). Participants read 11 low-predictability sentences of increasing length at their normal speaking rate and loudness. The sentences were digitally recorded, and then, the onsets and offsets of each sentence were marked and used in the calculation of the number of words spoken per minute by the SIT software. Speaking rate is the recommended measure for bulbar dysfunction; it has been associated with a relatively early and linear decline with disease progression [57] as well as the lesion loci in the UMNs [58].

#### 2.2.2. Cognitive Testing

A full neuropsychological battery composed of the North American Adult Reading Test (NAART) [59], the Judgment of Line Orientation (15-item) [60], Benton Facial Recognition (short-form) [61], Boston Naming Test (15-item) [62], and Digit Span Forward and Backward was performed on all participants (see report in [51]). However, for our further analyses, we chose to use a series of cognitive tasks within a novel computerized frontal battery (ALS-CFB) because in Gillingham et al.'s study, these tasks showed an improved sensitivity for detecting cognitive changes in ALS relative to the standard neuropsychological battery. The ALS-CFB included the following: (1) feature integration test (FIT), (2) emotion perception, (3) theory of mind, and (4) antisaccades.

*(1) Energization and Attentional Processes*. FIT, a reaction time test, measured energization (i.e., initiating and sustaining a response), task setting, and response monitoring processes [19]. During simple reaction time (SRT) task, participants were asked to press a response button as quickly as possible when they saw the target stimulus on a computer screen. The SRT task was run twice (SRT1 and SRT2), in the beginning and at the end of the FIT block. The response time (ms) in the SRT tasks was recorded in the condition of absent cognitive load. SRT1 was subtracted from the other RT tasks to adjust for differences in hand motor dysfunction. During easy reaction time (ERT) and complex reaction time (CRT) tasks, participants were presented with a relatively easy (i.e., one feature to monitor) or complex (i.e., more than one feature to monitor) target and nontarget stimuli (e.g., a geometric shape, color, and filler pattern). They were asked to press a response button 1 as quickly as possible when the target appeared on the screen and button 2 when any nontarget stimuli appeared. Reaction time was recorded electronically using the E-Prime 1.2 software [63]; errors were counted. Energization is the best reflected in the slowing of SRT2 (but is a contributor to all of the other tasks as well); task setting and task monitoring are reflected, in the absence of errors, by the increased slowing of ERT and CRT [24].

*(2) Emotional Processing*. Emotion perception abilities were tested using a 12-picture modified Ekman face set depicting four different emotions (i.e., happy, angry, sad, and neutral) [64]. The participants were shown a picture and the four words identifying the emotions and asked to press a response button when the correct word matching the picture was highlighted on the computer screen. The number of errors in identification of emotion was recorded.

*(3) Theory of Mind*. ToM test consisted of five false-belief first- and second-order cognitive reasoning stories and one affective story. The tasks were aimed at revealing one's mental ability to make inferences about thoughts, inexplicit actions, and feelings of others. All stories were presented on the computer screen and read orally to the participants. Two questions followed each story. The participants were asked to select one answer out of four multiple-choice responses, by pressing a button on the response box when the correct answer appeared. The total score (maximum of 12) was recorded, with a maximum score for the cognitive category being 10 and for the affective category being 2, across all stories.

*(4) Antisaccades*. Saccade recordings were obtained using custom-made software that allowed video recordings of eye movements via a build-in laptop camera [65]. Similar to the clinical saccades test, the participants were instructed to track a symbol (circle) on the computer screen from a fixation point (star), in pro- (1 block) and antisaccade (2 blocks) sequences. Twenty-four trials were recorded for each block. The total correct responses on both blocks of antisaccades (maximum score of 48) were measured for this study. The errors were manually counted during video analysis by a single rater; 20% of recordings were judged by a second independent rater with interrater reliability of 96.14%.

#### 2.2.3. MRI Acquisition and Postprocessing

A 3T Philips Achieva scanner was used to acquire the following: (1) a T1-weighted axial 3D FFE (1.99 ms TE, 25 ms TR, 30° flip angle, 24 × 24 × 13 cm FOV, 1 × 1 × 1 mm in plane resolution, 186 slices); an interleaved (2) proton density (PD); and (3) T2-weighted interleaved 2D axial dual turbo spin echo (11 and 102 ms TE, 2500 ms TR, 22.4 × 22.4 × 12 cm FOV, 1 × 1 × 1.5 mm in plane resolution, 54 slices). Volumetric measures were selected because they previously showed high sensitivity in detecting disease-related changes in ALS [66–68].

**Figure 1:**
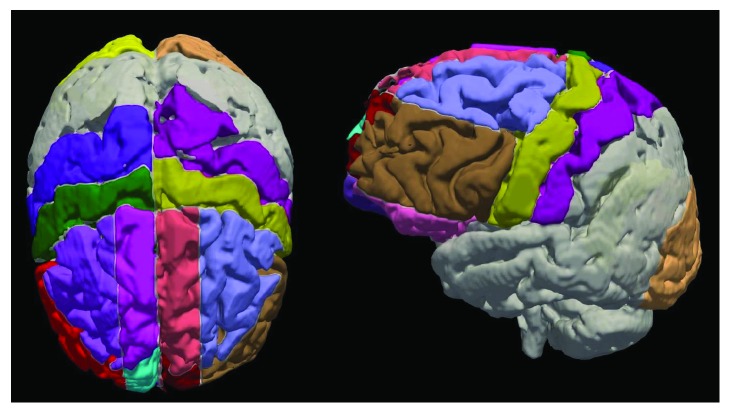
A 3D volume surface-rendered cerebral cortex showing the nine cortical regions used in the analyses with SABRE: axial view (left) and left hemisphere sagittal view (right). Regions of interest correspond to lateral superior, middle, and inferior frontal; medial superior, medial middle, and medial inferior frontal; precentral, postcentral, and occipital.

MRI-derived regional volumes for grey matter (GM), white matter (WM), cerebrospinal fluid (CSF), ventricular CSF (vCSF), and white matter hyperintensities of presumed vascular origin (WMH), as well as parcellation of specific regions, were obtained using a comprehensive, previously published, and rigorously validated image processing pipeline called SABRE [69–71]. This method is a trifeature (T1, T2, and PD) semiautomated segmentation algorithm that effectively segments each voxel into one of the five brain tissue classes (GM, WM, CSF, vCSF, and WMH) [72] and then parcellates each voxel into one of twenty-six different brain regions based on the Talairach proportional grid system [73, 74]. For our analyses, a modified version of SABRE was used, in which the pre- and postcentral gyri were identified by a highly trained operator and sulci boundaries were hand-traced. For this study, only the 6 frontal lobe regions (i.e., lateral superior, middle and inferior fronta, medial superior, medial middle, and medial inferior frontal), as well as the 2 sensorimotor regions (i.e., pre- and postcentral) per hemisphere were selected. These areas were chosen as they approximated the anatomical regions associated with the cognitive processes and tasks under study as well as the overall motor decline in ALS. Additionally, right and left occipital lobe regions served as controls in the analyses.  Figure 1 shows the 9 regions per hemisphere that were chosen for analyses.

The SABRE pipeline provided measures of GM and WM volumes within each region, as expressed as a percentage of regional volume, where regional volume is the equivalent proportion of total brain volume corrected for individual head size. In addition to GM and WM, CSF volumes were included in the analysis since the SABRE pipeline effectively segments subarachnoid CSF to account for intracranial variability. Image registration, brain extraction, ventricular assignment, and WMH segmentation were checked and/or edited manually for errors using ITK-SNAP software [75]. To date, this segmentation method has been shown to be sensitive to structural brain changes associated with normal aging, frontotemporal dementia, and traumatic brain injury [76–79].

### 2.3. Statistical Analyses

Analyses were conducted using IBM SPSS Statistics v. 19 (SPSS, 2010) (IBM Corp. Released 2010, IBM SPSS Statistics for Windows, Version 19.0. Armonk, NY: IBM Corp.). Group differences between patients with ALS and healthy controls in cognitive measures, including speaking rate and volumetric measures, were assessed using either the independent samples *t*-test or the Wilcoxon rank-sum test, depending on the normality of the data distribution, with a two-tailed *p*-value significance set at 0.05 (nonadjusted, due to the exploratory nature of the study). A partial least square regression method (PLS) was used to identify the neuroanatomical predictors of the behavioral (cognitive and speech rate) measures. PLS was selected because of the issue of multicollinearity between dependent variables. The variable importance in projection (VIP) and standardized parameter estimates (*β*) were identified for each model predicting behavioral (cognitive and motor) scores [80]. The VIP values reflected the importance of the latent variables with respect to *Y* (correlation to all the responses) and *X* (the projection). If a predictor had a relatively small coefficient (in absolute value) and a small value of VIP, it was removed from the model [81]. A two-step approach was used to provide greater accuracy to our final model. In the first iteration of PLS, all regions with VIP < 0.8, which is considered small, were removed from the models (SAS/STAT® User's Guide, 2011). Latent variables with VIP > 1, which signify a high association, were identified in the second iteration of the model and graphically examined along with their corresponding regression coefficients.

## 3. Results

### 3.1. Participant Description: Cognitive Profiles and Bulbar Motor Profiles

Descriptive statistics for the behavioral measures used in this study are displayed in Table 2. The measures that statistically distinguished ALS from the control group are marked with an asterisk. At a descriptive level, 59% (10/17) of patients showed performance < 1.5 SD of that expected in the control group on two or more cognitive tests. 35% (6/17) of patients exhibited impaired speaking rate.

### 3.2. Group Differences in Regional Volumes

Volumetric analysis revealed brain tissue atrophy in sensorimotor (pre- and postcentral) and extramotor frontal (i.e., lateral superior frontal and medial superior frontal) regions bilaterally in participants with ALS relative to controls (Table 3). Superior medial and superior frontal regions approximated the regions that are associated with the specific cognitive processes in the Stuss model.

### 3.3. Associations with Neuroanatomical Findings

**Figure 2:**
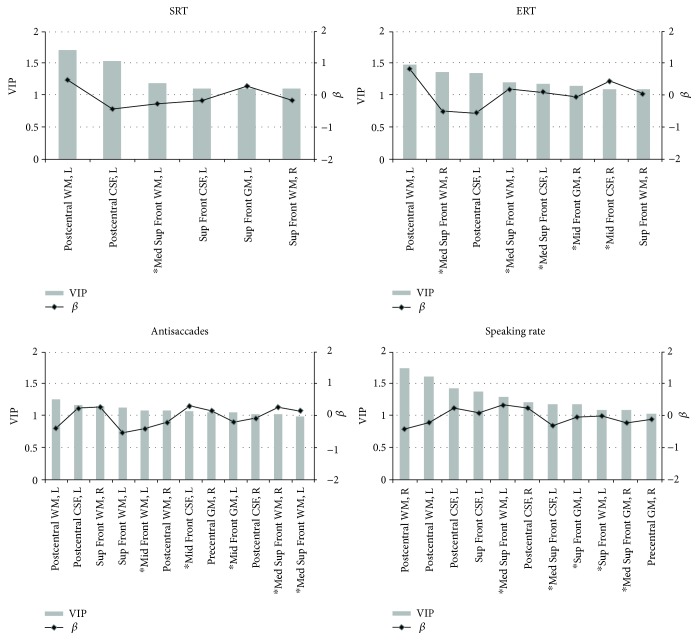
Results of the partial least square (PLS) regression analysis for selected behavioral measures. The SABRE brain volumetric predictors that supported our hypotheses based on Stuss' model [14] and speech production models are marked with an asterisk.

Figure 2 shows the results of the partial least square (PLS) regression analysis for the behavioral variables that differed significantly between the ALS and control groups. Only predictors with VIP > 1 (highly significant) are shown. The brain regions that showed an association with behavioral measures as predicted based on the model by Stuss [14] and those linked to speaking rate changes are marked with an asterisk on each plot.

Sensorimotor regions—precentral (primary motor cortex) and postcentral (somatosensory cortex)—as well as the superior frontal regions associated with supplementary and presupplementary motor areas were prominent across all PLS models, suggesting that the worsening performance on each task was associated with the volumetric changes in the regions supporting various motor aspects of the task execution. The volume reduction in the medial superior regions on the right and left, which according to Stuss [14] is responsible for energization, was also implicated in changes in performance on each of the examined PLS models.

Slowing of the SRT2 was associated—beyond the predicted medial superior frontal region—with changes only in the “motor” (the postcentral and superior frontal) regions. Slowing of ERT was associated, as predicted, with changes in the middle frontal gyrus (GM+CSF) on the right (task monitoring). In addition to the “motor” regions (pre- and postcentral and superior frontal) and the medial superior frontal regions bilaterally, the performance on antisaccades was linked to volumetric changes in the middle frontal region on the left, which contains DLPFC. The measure of speaking rate showed the highest associations, beyond the medial superior aspects on the left and right, with the “motor” regions including the superior frontal region on the left. Occipital (control) regions did not appear top-ranked in any of the four models.

## 4. Discussion

### 4.1. Summary of Results

There were three overarching findings in this study. First, the data supported our hypothesis that the distinct behavioral changes in cognition and speech production in ALS were related to specific regional neurostructural brain changes. Second, these behavioral changes did not seem to represent a general dysexecutive syndrome but were much more specific in nature. This specificity of the brain-behavior changes can provide a framework for subtyping of ALS patients and tracking the disease course in the future. Finally, the data supported a more integrative framework in the clinical assessment of frontal lobe functioning in ALS.

### 4.2. Brain-Behavior Associations in ALS

Our data supported the frontal lobe model [14] as means of understanding the neuroanatomical correlates of the frontal lobe deficits in ALS. Significant associations existed between the impairment in the specific cognitive processes tested via ALS-CFB [51] and tasks (e.g., antisaccades and speaking rate) and neurostructural changes in distinct regions of the frontal lobes.

#### 4.2.1. Energization and Executive Function

Patients with ALS in our study exhibited significant differences in energization (SRT2) and attention regulation (ERT) tasks. As predicted by the works of Stuss et al. based on data on patents with focal lesions [19, 23], the SRT2 was associated with volumetric changes in the white matter of the medial superior region on the left, while a more complex ERT task involved atrophy in the medial superior regions bilaterally and the middle frontal gyri on the right. Unsurprisingly, the remaining regions were primary and secondary association aspects of the motor system comprising the primary motor cortex, somatosensory cortex, and superior frontal regions containing supplementary and presupplementary motor cortex.

A small number of neuroimaging studies reported a link between attentional deficit and structural changes in the frontal lobes in ALS a[82, 83]. An fMRI study of verbal fluency, which involves similar attentional processes, indicated impaired frontal activation in the middle and inferior frontal gyri as well as the anterior cingulate [84], while a study of the Stroop task did not show a clear pattern of change in activation in the frontal lobes of patients with ALS [6]. These neuropsychological tests are complex in nature and tap into global cognitive domains rather than processes; examining distinctive processes might provide a clearer inference to the potential brain involvement in patients with ALS.

#### 4.2.2. Antisaccades

A previous research indicated that the performance on the antisaccade task is highly dependent on the integrity of multiple regions in the frontal lobes [21, 22, 85]. In this study, the degraded performance on the antisaccades was linked to volume reduction in the lateral superior and middle frontal regions and medial superior frontal regions, encompassing DLPFC, frontal eye fields, and supplementary and presupplementary motor cortices, but not the inferior frontal gyrus. A recent fMRI study documented an increase in the number of errors in patients with ALS on the antisaccade task, which was related to degraded activation in DLPFC, but compensatory responses in the presupplementary area and frontal eye fields [45]. A more detailed analysis of tissue atrophy in these ROIs would be necessary to examine the extent of damage in these regions relative to the compensatory response.

#### 4.2.3. Speaking Rate

Speaking rate as a measure of bulbar motor deficit in ALS was important to consider because it has been described as part of the overall cognitive-linguistic assessment in a number of frontal lobe aetiologies [49] including aphasia [86, 87]. In our sample, however, the slowing of the speaking rate was not associated with any extramotor regions beyond the superior medial frontal region responsible for energization. In ALS, bulbar deficit indexed by the ALSFRS-R bulbar subscore has been associated with cortical thinning in the ventral precentral gyrus (motor) but not to the extramotor regions in the past [88]. Combined, these results support linking speaking rate abnormalities to spastic (UMN) dysarthric (motor) deficits in ALS and not to a cognitive deficit [58]. In patients with primary progressive aphasia, slowing of the speaking rate has been associated with changes in the inferior frontal lobe, which includes Broca's area, as well as to the “motor” areas on the left, possibly indicating a higher level of processing deficit in PPA [48, 89].

Interestingly, changes across all tasks were associated with volumetric changes in the primary and secondary/association motor areas such as the primary motor cortex, somatosensory cortex, and superior frontal regions approximating the supplementary and presupplementary motor regions. The involvement of the medial superior regions was also noteworthy across all tasks as the effect of its regional changes on task execution, including speech, is well documented [90, 91]. There was no association, however, recorded between slowing on the RT and slowing in speech, suggesting different mechanisms underlying these tasks.

### 4.3. Is There Evidence for a General Dysexecutive Deficit in ALS?

Understanding of the frontal lobe functions in health and disease is challenging due to their structural and functional complexity. Opposing ideas—from independent fractionalized processes to united (e.g., “central executive”) constructs—are proposed to create a framework in which frontal lobes can be studied. Here, we acquired evidence supporting a fractionated view of the frontal lobes [14]. The strongest evidence for this view comes from the observation of disassociations between various cognitive processes. When results were compared across different ALS-CFB subtests, nine patients exhibited deficits on the SRT2 task (adjusted for motor slowness), while only three of these patients also exhibited difficulties on the ERT task, while 11 patients showed impaired antisaccades. Relative to SRT, antisaccades were uniquely impaired in 4 patients. Clearly, different patients can exhibit specific frontal lobe deficits, which may uniquely reflect the pattern of frontal lobe neurodegeneration.

Additionally, cognitive scores were independent of the overall disease severity as measured by the ALSFRS-R total scores, suggesting the lack of the relationship between the overall motor disease state and cognitive performance. Similar results have been reported in the past [92, 93]. The severity of the bulbar motor disease measured by either the ALSFRS-R bulbar subscore or speaking rate did not correlate with the performance on cognitive tasks either.

Specificity of the cognitive behavioral changes emerging in ALS may provide a framework for further subtyping of ALS patients and tracking the course of the disease, beyond symptoms at onset (e.g., bulbar versus spinal). At present, the subtypes of (a) purely motor ALS, (b) ALS with cognitive impairment (ALSci), (c) ALS with behavioral impairment (ALSbi), and (d) ALS-FTD are relatively well accepted [94, 95]. However, evidence is emerging for subcategories of ALSci such as ALS with executive dysfunction (ALS-Ex) and ALS with language or memory deficits (ALS-NECI) [96]. The importance of establishing these categories is in the potential differences in the disease progression rates (e.g., a more aggressive disease in ALS-ex) and clinical prognostication. Neuroimaging validation of these behavioral categories is pending; however, our preliminary findings support the existence of specific neurostructural constructs for subcategories of cognitive impairment within the frontal lobes at a possibly even more fine-graded level.

### 4.4. Improving Assessment in ALS

With improved understanding of the nature of ALS, new instruments can emerge for screening cognitive/behavioral deficits in this clinical population (see [84, 97]). Our ALS-CFB is an example of the cognitive testing procedure that is process-oriented instead of domain/function oriented, provides anatomic specificity, is efficient in administration, and requires minimal motor responses [51]. It appears sensitive to frontal lobe deficits in patients with ALS; a computerized antisaccade task—a relatively simple and quick test—may also be highly useful in the assessment of frontal lobe health in ALS. A body of literature on improving detection and tracking progression of bulbar ALS is also emerging. Here, we provided a neurostructural support for speaking rate as part of the bulbar motor assessment. However, in the future, articulatory rate (i.e., rate computed with pauses removed) as well as percent pause measures should be considered because of their ability to detect changes earlier in the disease process than the speaking rate [98].

### 4.5. Study Limitations

The findings of this study were limited by several factors. First, small sample size limited the analyses we could perform in terms of determining group differences in neurostructural and cognitive measures. Moreover, more/other predictors could have reached the significance criterion should the sample size be larger. Second, the current version of ALS-CFB may have been limited in its ability to detect differences in other frontal lobe processes such as emotion recognition and ToM, restricting the extent of our correlational analyses. A short test length might have contributed to the nearly normal performance in our patient group and limited range of variability among participants with ALS in emotion recognition (see [51]). Refining the test to improve its ability to detect cognitive abnormalities, prevent learning effects, and delineate performance range would allow us to better evaluate the relationships between different cognitive processes and their neurostructural correlates. Finally, the choice of our imaging analyses may have had a significant effect on our findings. Structural volumetric changes with disease progression have been assessed for the first time using the SABRE pipeline. Its advantage is in being able to measure changes not only at the cortical level but also subcortically but, as a limitation, it only approximated our ROIs. In the future, analyses focused on specific ROIs are indicated.

## 5. Future Directions

Further work is needed on the development of the ALS-CFB as it offers a unique approach towards process-oriented testing in neuropsychology. Because cognitive performance can be described with higher specificity using the process-oriented point of view, a more refined understanding of cognitive performance and of individual variability can be achieved. The imaging work should be extended towards better understanding of cortical and corresponding white matter changes using other imaging modalities (e.g., DTI). In the future, it would be important to perform a whole-brain voxel-wise WM analysis looking at fractional anisotropy (FA) and mean diffusivity (MD) in ALS relative to healthy controls and then test their role in predicting the behavioral scores using the PLS model. Examination of networks associated with various functions and their selected vulnerability in the disease as a whole or individually may be the next step in the work on determining clinically relevant behavior-brain relationships.

## Figures and Tables

**Table 1:** Demographic and clinical information (group means, standard deviations); ALSFRS: ALS Functional Rating Scale-Revised; UMN: upper motor neuron.

	ALS (*N* = 17)	Controls (*N* = 12)
Age (yrs)	61.88, 8.92	62.25, 7.96
Education (yrs)	16.20, 2.86	16.25, 1.82
ALS duration, mns	34.41, 20.07	—
ALSFRS, total (/48)	39.70, 5.35	—
ALSFRS, bulbar (/12)	10.7, 1.70	—
UMN score (/10)	3.59, 1.54	—

**Table 2:** Summary statistics (mean, standard deviations) for cognitive measures and speaking rate by group. The asterisk indicates measures that showed a significant group difference (*p* < 0.05) between participants with ALS and controls. SRT: simple RT; ERT: easy RT; CRT: complex RT; ToM: theory of mind.

	ALS (*N* = 17)	Controls (*N* = 12)
SRT1	344.05, 64.49	278.81, 45.55
SRT2^∗^	416.55. 128.15	280.07, 39.69
ERT^∗^	647.74, 109.07	556.89, 60.64
CRT	725.14, 113.65	651.43, 102.71
Antisaccades^∗^	32.83, 9.87	42.25, 2.87
Emotion perception	10.06, 1.57	10.78, 1.30
ToM, cognitive	8.67, 1.72	9.88, 0.35
ToM, affective	1.67, 0.49	1.50 0.67
Speaking rate^∗^	168.02, 37.23	189.06, 29.27

**Table 3:** Comparisons of SABRE-generated regional volumes by tissue class between patients with ALS and controls. Only statistically significant results are presented (df = 27). SD: standard deviation; GM: grey matter; WM: white matter; CSF: cerebrospinal fluid; L: left; R: right.

Region, tissue class	ALS (mean, SD)	Controls (mean, SD)	*W* statistic	*p* value
Precentral, WM, L	36.21, 4.65	41.44, 5.19	236	0.020
Precentral, CSF, L	23.46, 6.45	17.45, 4.37	119	0.012
Precentral, CSF, R	23.17, 7.21	17.28, 2.75	123	0.019
Postcentral, CSF, L	22.17, 5.54	17.67, 2.73	130	0.037
Postcentral, GM, R	37.53, 3.87	40.14, 2.52	228	0.045
Superior frontal, GM, L	46.18, 5.84	52.62, 2.80	258	0.002
Superior frontal, WM,L	20.40, 4.31	24.05, 4.16	227	0.049
Superior frontal, CSF, L	33.40, 8.31	23.32, 4.54	107	0.003
Superior frontal, GM, R	46.12, 7.99	51.43, 3.46	240	0.014
Superior frontal, WM, R	19.33, 7.80	23.80, 3.08	228	0.045
Superior frontal, CSF, R	34.55, 12.00	24.76, 3.94	125	0.023
Medial Sup frontal, GM, L	40.10, 5.65	44.78, 3.31	229	0.041
Medial Sup frontal, WM, R	21.20, 5.76	25.57, 3.10	231	0.034
Medial Sup frontal, CSF, R	36.84, 10.40	28.52, 4.67	130	0.037

## Data Availability

The data used to support the findings of this study are available from the corresponding author upon request and competion of required data sharing agreements

## Additional Points

*Highlights*. Distinctive behavioral changes in patients with ALS may be related to distinctive neurostructural changes. The patterns of these changes do not seem to support the notion of the generalized dysexecutive syndrome in ALS.

## References

[1] Strong M. J., Grace G. M., Freedman M. (2009). Consensus criteria for the diagnosis of frontotemporal cognitive and behavioural syndromes in amyotrophic lateral sclerosis. *Amyotrophic Lateral Sclerosis*.

[2] Abrahams S., Goldstein L. H., Kew J. J. M. (1996). Frontal lobe dysfunction in amyotrophic lateral sclerosis. *Brain*.

[3] del Aguila M. A., Longstreth W. T., McGuire V., Koepsell T. D., van Belle G. (2003). Prognosis in amyotrophic lateral sclerosis a population-based study. *Neurology*.

[4] Schreiber H., Gaigalat T., Wiedemuth-Catrinescu U. (2005). Cognitive function in bulbar- and spinal-onset amyotrophic lateral sclerosis. A longitudinal study in 52 patients. *Journal of Neurology*.

[5] Goldstein L. H., Abrahams S. (2013). Changes in cognition and behaviour in amyotrophic lateral sclerosis: nature of impairment and implications for assessment. *The Lancet Neurology*.

[6] Goldstein L. H., Newsom-Davis I. C., Bryant V., Brammer M., Leigh P. N., Simmons A. (2011). Altered patterns of cortical activation in ALS patients during attention and cognitive response inhibition tasks. *Journal of Neurology*.

[7] Phukan J., Pender N. P., Hardiman O. (2007). Cognitive impairment in amyotrophic lateral sclerosis. *The Lancet Neurology*.

[8] Tsermentseli S., Leigh P. N., Goldstein L. H. (2012). The anatomy of cognitive impairment in amyotrophic lateral sclerosis: more than frontal lobe dysfunction. *Cortex*.

[9] Jurado M. B., Rosselli M. (2007). The elusive nature of executive functions: a review of our current understanding. *Neuropsychology Review*.

[10] Norman D. A., Shallice T. (1980). *Attention to Action: Willed and Automatic Control of Behavior*.

[11] Burgess P. W., Stuss D. T. (2017). Fifty years of prefrontal cortex research: impact on assessment. *Journal of the International Neuropsychological Society*.

[12] Shallice T., Stuss D. T., Alexander M. P., Picton T. W., Derkzen D. (2008). The multiple dimensions of sustained attention. *Cortex*.

[13] Stuss D. T., Alexander M. P. (2007). Is there a dysexecutive syndrome?. *Philosophical Transactions of the Royal Society*.

[14] Stuss D. T. (2011). Functions of the frontal lobes: relation to executive functions. *Journal of the International Neuropsychological Society*.

[15] Stuss D. T., Levine B. (2002). Adult clinical neuropsychology: lessons from studies of the frontal lobes. *Annual Review of Psychology*.

[16] Floden D., Vallesi A., Stuss D. T. (2011). Task context and frontal lobe activation in the Stroop task. *Journal of Cognitive Neuroscience*.

[17] Picton T. W., Stuss D. T., Alexander M. P., Shallice T., Binns M. A., Gillingham S. (2006). Effects of focal frontal lesions on response inhibition. *Cerebral Cortex*.

[18] Stuss D. T., Floden D., Alexander M. P., Levine B., Katz D. (2001). Stroop performance in focal lesion patients: dissociation of processes and frontal lobe lesion location. *Neuropsychologia*.

[19] Stuss D. T., Alexander M. P., Floden D., Stuss D. T., Knight R. T. (2002). Fractionation and localization of distinct frontal lobe processes: evidence from focal lesions in humans. *Principles of Frontal Lobe Function*.

[20] Stuss D. T., Gallup G., Alexander M. P. (2001). The frontal lobes are necessary for ‘theory of mind’. *Brain*.

[21] Munoz D. P., Coe B. C. (2011). Saccade, search and orient- the neural control of saccadic eye movements. *European Journal of Neuroscience*.

[22] Wiecki T. V., Frank M. J. (2013). A computational model of inhibitory control in frontal cortex and basal ganglia. *Psychological Review*.

[23] Shallice T., Stuss D. T., Picton T. W., Alexander M. P., Gillingham S. (2008). Multiple effects of prefrontal lesions on task-switching. *Frontiers in Human Neuroscience*.

[24] Stuss D. T., Alexander M. P., Shallice T. (2005). Multiple frontal systems controlling response speed. *Neuropsychologia*.

[25] Stuss D. T., Binns M. A., Murphy K. J., Alexander M. P. (2002). Dissociation within the anterior attentional system: effects of task complexity and irrelevant information on reaction time speed and accuracy. *Neuropsychology*.

[26] Vallesi A., McIntosh A. R., Crescentini C., Stuss D. T. (2012). fMRI investigation of speed–accuracy strategy switching. *Human Brain Mapping*.

[27] Vallesi A., McIntosh A. R., Shallice T., Stuss D. T. (2009). When time shapes behavior: fMRI evidence of brain correlates of temporal monitoring. *Journal of Cognitive Neuroscience*.

[28] Mannarelli D., Pauletti C., Locuratolo N. (2014). Attentional processing in bulbar-and spinal-onset amyotrophic lateral sclerosis: insights from event-related potentials. *Amyotrophic Lateral Sclerosis and Frontotemporal Degeneration*.

[29] Pinkhardt E. H., Jurgens R., Becker W. (2008). Signs of impaired selective attention in patients with amyotrophic lateral sclerosis. *Journal of Neurology*.

[30] Kasper E., Schuster C., Machts J. (2015). Dysexecutive functioning in ALS patients and its clinical implications. *Amyotrophic Lateral Sclerosis and Frontotemporal Degeneration*.

[31] Burke T., Elamin M., Bede P. (2016). Discordant performance on the ‘Reading the Mind in the Eyes’ test, based on disease onset in amyotrophic lateral sclerosis. *Amyotrophic Lateral Sclerosis and Frontotemporal Degeneration*.

[32] Girardi A., Macpherson S. E., Abrahams S. (2011). Deficits in emotional and social cognition in amyotrophic lateral sclerosis. *Neuropsychology*.

[33] Papps B., Abrahams S., Wicks P., Leigh P. N., Goldstein L. H. (2005). Changes in memory for emotional material in amyotrophic lateral sclerosis (ALS). *Neuropsychologia*.

[34] Schmolck H., Mosnik D., Schulz P. (2007). Rating the approachability of faces in ALS. *Neurology*.

[35] Zimmerman E. K., Eslinger P. J., Simmons Z., Barrett A. M. (2007). Emotional perception deficits in amyotrophic lateral sclerosis. *Cognitive and Behavioral Neurology*.

[36] Meier S. L., Charleston A. J., Tippett L. J. (2010). Cognitive and behavioural deficits associated with the orbitomedial prefrontal cortex in amyotrophic lateral sclerosis. *Brain*.

[37] van der Hulst E.-J., Bak T. H., Abrahams S. (2014). Impaired affective and cognitive theory of mind and behavioural change in amyotrophic lateral sclerosis. *Journal of Neurology, Neurosurgery & Psychiatry*.

[38] Donaghy C., Pinnock R., Abrahams S. (2010). Slow saccades in bulbar-onset motor neurone disease. *Journal of Neurology*.

[39] Evdokimidis I., Constantinidis T. S., Gourtzelidis P. (2002). Frontal lobe dysfunction in amyotrophic lateral sclerosis. *Journal of the Neurological Sciences*.

[40] Shaunak S., Orrell R. W., O'Sullivan E. (1995). Oculomotor function in amyotrophic lateral sclerosis: evidence for frontal impairment. *Annals of Neurology*.

[41] Cerami C., Dodich A., Canessa N. (2014). Emotional empathy in amyotrophic lateral sclerosis: a behavioural and voxel-based morphometry study. *Amyotrophic Lateral Sclerosis and Frontotemporal Degeneration*.

[42] Crespi C., Cerami C., Dodich A. (2014). Microstructural white matter correlates of emotion recognition impairment in amyotrophic lateral sclerosis. *Cortex*.

[43] Kasper E., Schuster C., Machts J. (2014). Microstructural white matter changes underlying cognitive and behavioural impairment in ALS–an in vivo study using DTI. *PLoS One*.

[44] Sarro L., Agosta F., Canu E. (2011). Cognitive functions and white matter tract damage in amyotrophic lateral sclerosis: a diffusion tensor tractography study. *American Journal of Neuroradiology*.

[45] Witiuk K., Fernandez-Ruiz J., McKee R. (2014). Cognitive deterioration and functional compensation in ALS measured with fMRI using an inhibitory task. *The Journal of Neuroscience*.

[46] Bohland J. W., Guenther F. H. (2006). An fMRI investigation of syllable sequence production. *NeuroImage*.

[47] Guenther F. H., Ghosh S. S., Tourville J. A. (2006). Neural modeling and imaging of the cortical interactions underlying syllable production. *Brain and Language*.

[48] Wilson S. M., Henry M. L., Besbris M. (2010). Connected speech production in three variants of primary progressive aphasia. *Brain*.

[49] Stuss D. T., Benson D. F. (1986). *The Frontal Lobes*.

[50] Ball L. J., Willis A., Beukelman D. R., Pattee G. L. (2001). A protocol for identification of early bulbar signs in amyotrophic lateral sclerosis. *Journal of the Neurological Sciences*.

[51] Gillingham S. M., Yunusova Y., Ganda A. (2017). Assessing cognitive functioning in ALS: a focus on frontal lobe processes. *Amyotrophic Lateral Sclerosis and Frontotemporal Degeneration*.

[52] Flinker A., Korzeniewska A., Shestyuk A. Y. (2015). Redefining the role of Broca’s area in speech. *Proceedings of the National Academy of Sciences*.

[53] Brooks B. R., Miller R. G., Swash M., Munsat T. L. (2009). El Escorial revisited: revised criteria for the diagnosis of amyotrophic lateral sclerosis. *Amyotrophic Lateral Sclerosis and Other Motor Neuron Disorders*.

[54] Nasreddine Z. S., Phillips N. A., Bédirian V. (2005). The Montreal Cognitive Assessment, MoCA: a brief screening tool for mild cognitive impairment. *Journal of the American Geriatrics Society*.

[55] Cedarbaum J. M., Stambler N., Malta E. (1999). The ALSFRS-R: a revised ALS functional rating scale that incorporates assessments of respiratory function. BDNF ALS study group (phase III). *Journal of the Neurological Sciences*.

[56] Yorkston K. M., Beukelman D., Hakel M., Dorsey M. (2007). *Sentence Intelligibility Test*.

[57] Yorkston K. M. (1993). Speech deterioration in amyotrophic lateral sclerosis: implications for the timing of intervention. *Jounal of Medical Speech-Language Pathology*.

[58] Duffy J. R. (2005). *Motor Speech Disorders: Substrates, Differential Diagnosis, and Management*.

[59] Blair J. R., Spreen O. (1989). Predicting premorbid IQ: a revision of the National Adult Reading Test. *The Clinical Neuropsychologist*.

[60] Benton A. L., Varney N. R., Hamsher K. D. (1978). Visuospatial judgement: A clinical test. *Archives of Neurology*.

[61] Ekman P. (1976). *Pictures of Facial Affect*.

[62] Kaplan E., Goodglass H., Weintraub S. (1983). *The Boston Naming Test*.

[63] Schneider W., Eschman A., Zuccolotto A. (2012). *E-Prime: User’s Guide*.

[64] Ekman P., Friesen W. V. (1976). Measuring facial movement. *Environmental Psychology and Nonverbal Behavior*.

[65] Kaufman L. D., Pratt J., Levine B., Black S. E. (2010). Antisaccades: a probe into the dorsolateral prefrontal cortex in Alzheimer’s disease. A critical review. *Journal of Alzheimer's Disease*.

[66] Grosskreutz J., Kaufmann J., Frädrich J., Dengler R., Heinze H.-J., Peschel T. (2006). Widespread sensorimotor and frontal cortical atrophy in amyotrophic lateral sclerosis. *BMC Neurology*.

[67] Kassubek J., Unrath A., Huppertz H.‐. J. (2009). Global brain atrophy and corticospinal tract alterations in ALS, as investigated by voxel-based morphometry of 3-D MRI. *Amyotrophic Lateral Sclerosis and Other Motor Neuron Disorders*.

[68] Turner M. R., Hammers A., Allsop J. (2009). Volumetric cortical loss in sporadic and familial amyotrophic lateral sclerosis. *Amyotrophic Lateral Sclerosis*.

[69] Ramirez J., Gibson E., Quddus A. (2011). Lesion explorer: a comprehensive segmentation and parcellation package to obtain regional volumetrics for subcortical hyperintensities and intracranial tissue. *NeuroImage*.

[70] Ramirez J., Scott C. J. M., Black S. E. (2013). A short-term scan-rescan reliability test measuring brain tissue and subcortical hyperintensity volumetrics obtained using the lesion explorer structural MRI processing pipeline. *Brain Topography*.

[71] Ramirez J., Scott C. J., McNeely A. A. (2014). Lesion explorer: a video-guided, standardized protocol for accurate and reliable MRI-derived volumetrics in Alzheimer’s disease and normal elderly. *Journal of Visualized Experiments*.

[72] Kovacevic N., Lobaugh N. J., Bronskill M. J., Levine B., Feinstein A., Black S. E. (2002). A robust method for extraction and automatic segmentation of brain images. *NeuroImage*.

[73] Dade L. A., Gao F. Q., Kovacevic N. (2004). Semiautomatic brain region extraction: a method of parcellating brain regions from structural magnetic resonance images. *NeuroImage*.

[74] Levy-Cooperman N., Ramirez J., Lobaugh N. J., Black S. E. (2008). Misclassified tissue volumes in Alzheimer disease patients with white matter hyperintensities importance of lesion segmentation procedures for volumetric analysis. *Stroke*.

[75] Yushkevich P. (2006). ITK-SNaP integration, NLM insight. http://www.itk.org/index.

[76] Bocti C., Rockel C., Roy P., Gao F., Black S. E. (2006). Topographical patterns of lobar atrophy in frontotemporal dementia and Alzheimer’s disease. *Dementia and Geriatric Cognitive Disorders*.

[77] Chow T. W., Binns M. A., Freedman M. (2008). Overlap in frontotemporal atrophy between normal aging and patients with frontotemporal dementias. *Alzheimer Disease and Associated Disorders*.

[78] Ramirez J., McNeely A. A., Scott C. J., Stuss D. T., Black S. E. (2014). Subcortical hyperintensity volumetrics in Alzheimer’s disease and normal elderly in the Sunnybrook Dementia Study: correlations with atrophy, executive function, mental processing speed, and verbal memory. *Alzheimer's Research & Therapy*.

[79] Söderlund H., Black S. E., Miller B. L., Freedman M., Levine B. (2008). Episodic memory and regional atrophy in frontotemporal lobar degeneration. *Neuropsychologia*.

[80] Chong I.-G., Jun C.-H. (2005). Performance of some variable selection methods when multicollinearity is present. *Chemometrics and Intelligent Laboratory Systems*.

[81] Wold S., Sjöström M., Eriksson L. (2001). PLS-regression: a basic tool of chemometrics. *Chemometrics and Intelligent Laboratory Systems*.

[82] Abe K., Fujimura H., Toyooka K., Sakoda S., Yorifuji S., Yanagihara T. (1997). Cognitive function in amyotrophic lateral sclerosis. *Journal of Neurological Sciences*.

[83] Evans J., Olm C., McCluskey L. (2015). Impaired cognitive flexibility in amyotrophic lateral sclerosis. *Cognitive And Behavioral Neurology*.

[84] Abrahams S., Newton J., Niven E., Foley J., Bak T. H. (2014). Screening for cognition and behaviour changes in ALS. *Amyotrophic Lateral Sclerosis and Frontotemporal Degeneration*.

[85] Erika-Florence M., Leech R., Hampshire A. (2014). A functional network perspective on response inhibition and attentional control. *Nature Communications*.

[86] Perez D. L., Dickerson B. C., McGinnis S. M. (2013). You don’t say: dynamic aphasia, another variant of primary progressive aphasia?. *Journal of Alzheimer's Disease*.

[87] Robinson G., Blair J., Cipolotti L. (1998). Dynamic aphasia: an inability to select between competing verbal responses?. *Brain*.

[88] Schuster C., Kasper E., Dyrba M. (2014). Cortical thinning and its relation to cognition in amyotrophic lateral sclerosis. *Neurobiology of Aging*.

[89] Ash S., Moore P., Vesely L. (2009). Non-fluent speech in frontotemporal lobar degeneration. *Journal of Neurolinguistics*.

[90] Alario F.-X., Chainay H., Lehericy S., Cohen L. (2006). The role of the supplementary motor area (SMA) in word production. *Brain Research*.

[91] Ziegler W., Kilian B., Deger K. (1997). The role of the left mesial frontal cortex in fluent speech: evidence from a case of left supplementary motor area hemorrhage. *Neuropsychologia*.

[92] Flaherty-Craig C., Brothers A., Dearman B., Eslinger P., Simmons Z. (2009). Penn State screen exam for the detection of frontal and temporal dysfunction syndromes: application to ALS. *Amyotrophic Lateral Sclerosis*.

[93] Murphy J., Factor-Litvak P., Goetz R. (2016). Cognitive-behavioral screening reveals prevalent impairment in a large multicenter ALS cohort. *Neurology*.

[94] Lomen-Hoerth C., Strong M. J. (2005). *Frontotemporal Dysfunction in Amyotrophic Lateral Sclerosis Amyotrophic Lateral Sclerosis*.

[95] Murphy J., Henry R., Lomen-Hoerth C. (2007). Establishing subtypes of the continuum of frontal lobe impairment in amyotrophic lateral sclerosis. *Archives of Neurology*.

[96] Phukan J., Elamin M., Bede P. (2011). The syndrome of cognitive impairment in amyotrophic lateral sclerosis: a population-based study. *Journal of Neurology, Neurosurgery and Psychiatry*.

[97] Woolley S. C., York M. K., Moore D. H. (2010). Detecting frontotemporal dysfunction in ALS: utility of the ALS Cognitive Behavioral Screen (ALS-CBS). *Amyotrophic Lateral Sclerosis*.

[98] Allison K. M., Yunusova Y., Campbell T. F., Wang J., Berry J. D., Green J. R. (2017). The diagnostic utility of patient-report and speech-language pathologists’ ratings for detecting the early onset of bulbar symptoms due to ALS. *Amyotrophic Lateral Sclerosis and Frontotemporal Degeneration*.

